# Transgenic animal models of congenital diaphragmatic hernia: a comprehensive overview of candidate genes and signaling pathways

**DOI:** 10.1007/s00383-020-04705-0

**Published:** 2020-06-26

**Authors:** Hiroki Nakamura, Takashi Doi, Prem Puri, Florian Friedmacher

**Affiliations:** 1 National Children’s Research Centre, Our Lady’s Children’s Hospital, Dublin, Ireland; 2 Department of Pediatric Surgery, Kansai Medical University, Osaka, Japan; 3 Beacon Hospital, University College Dublin, Dublin, Ireland; 4 Department of Pediatric Surgery, University Hospital Frankfurt, Goethe University Frankfurt, Theodor-Stern-Kai 7, 60590 Frankfurt am Main, Germany

**Keywords:** Congenital diaphragmatic hernia, Pulmonary hypoplasia, Lung development, Pulmonary hypertension, Transgenic mice, Retinoic acid

## Abstract

Congenital diaphragmatic hernia (CDH) is a relatively common and life-threatening birth defect, characterized by incomplete formation of the diaphragm. Because CDH herniation occurs at the same time as preacinar airway branching, normal lung development becomes severely disrupted, resulting almost invariably in pulmonary hypoplasia. Despite various research efforts over the past decades, the pathogenesis of CDH and associated lung hypoplasia remains poorly understood. With the advent of molecular techniques, transgenic animal models of CDH have generated a large number of candidate genes, thus providing a novel basis for future research and treatment. This review article offers a comprehensive overview of genes and signaling pathways implicated in CDH etiology, whilst also discussing strengths and limitations of transgenic animal models in relation to the human condition.

## Introduction

Congenital diaphragmatic hernia (CDH) is a developmental abnormality characterized by the presence of a defect in the integrity of the forming diaphragm, affecting between 1.9 and 2.3 cases per 10,000 newborns in the United States [1] and Europe [2]. Defects in the posterolateral diaphragm, commonly referred to as Bochdalek hernias, comprise approximately 90-95% of all CDH cases with about 80% occurring on the left side, 15% on the right and less than 5% bilaterally [3]. Non-posterolateral CDH manifests as anterior conditions such as Morgagni hernias in the anterior retrosternal or peristernal diaphragm and central hernias in the central tendinous portion of the diaphragm [4]. Posterolateral diaphragmatic defects permit protrusion of the abdominal viscera into the thoracic cavity, thus interfering with normal lung development and frequently leading to severe respiratory distress at birth due to the unfortunate combination of pulmonary hypoplasia and persistent pulmonary hypertension of the newborn [3, 5].

Over the last decade, CDH remained a life-threatening congenital disorder with mortality rates up to 50% [6–9]. Treatment usually consists of surgical movement of the abdominal viscera out of the thoracic cavity and closure of the diaphragmatic defect. Large defects may be difficult to repair through direct sutures, requiring the use of a prosthetic patch or abdominal muscle flap [10, 11]. Apart from surgical methods, treatment options for CDH are limited due to its poorly understood etiology, thereby motivating the need for better experimental models to elucidate its pathogenesis while also testing new therapeutic approaches. Investigation of novel medical therapies and pharmacological compounds that have the ability to arrest or reverse associated lung hypoplasia in animal models of CDH require the application of standardized research methodologies [12]. In this review article, we discuss the development of and findings associated with transgenic animal models of CDH to highlight the progress made to date in understanding CDH pathogenesis and evolution.

### Transgenic animal models of CDH

Both environmental and genetic factors are thought to contribute to the etiology of CDH. To date, genetic causes have been identified in approximately 30% of neonates with CDH [13–15]. With the advent of innovative molecular techniques in recent years, transgenic animal models of CDH have become more common, offering new candidate genes and signaling pathways implicated in the pathogenesis and etiology of diaphragmatic defects and associated lung abnormalities (Table 1). So far, 18 mouse models with phenotypic similarities to human CDH have been listed in the Mouse Genome Database (http://www.informatics.jax.org).

**Table 1:** Transgenic mouse models of congenital diaphragmatic hernia

Symbol	Gene name	Diaphragmatic defect	Pulmonary abnormality
*Rarα, Rarβ2*	Retinoic acid receptor, α and β2	Diaphragmatic hernia	Lung hypoplasia, abnormal alveoli
*Lrp1*	Low density lipoprotein receptor-related protein 1	Diaphragmatic hernia	–
*Nr2f2 (Couptf2)*	Nuclear receptor subfamily 2, group F, member 2	Posterior diaphragmatic hernia	Lung hypoplasia
*Wt1*	Wilms tumor 1 homolog	Posterior diaphragmatic hernia	Lung hypoplasia
*Msc (MyoR)*	Musculin	Posterior diaphragmatic hernia	Lung hypoplasia, abnormal branching, abnormal vasculature
*Tcf21 (capsulin)*	Transcription factor 21	Posterior diaphragmatic hernia	Lung hypoplasia, abnormal branching, abnormal vasculature
*Gli2/3*	GLI-Kruppel family member 2 and 3	Diaphragmatic hernia	Lung hypoplasia, absent right lung accessory lobe, thick mesenchyme
*Shh*	Sonic hedgehog	Diaphragmatic hernia	Lung hypoplasia
*Kif7*	Kinesin family member 7	Posterior diaphragmatic hernia	Lung hypoplasia
*Zfpm2 (Fog2)*	Zinc finger protein, multitype 2	Posterolateral diaphragmatic hernia	Lung hypoplasia, absent right lung accessory lobe
*Gata4*	GATA-binding protein 4	Central diaphragmatic hernia (with sac)	Abnormal saccule morphologic features, abnormal vasculature
*Sox7*	Sex determining region Y-box 7	Retrosternal diaphragmatic hernia	–
*Frem1*	FRAS1-related extracellular matrix 1	Retrosternal diaphragmatic hernia	Lung lobulation defects
*c-Met*	Mesenchymal-epithelial transition factor	Amascular diaphragm with hernia	Abnormal saccule morphologic features
*Fgf10*	Fibroblast growth factor 10	Posterolateral diaphragmatic hernia	Lung hypoplasia
*Slit3*	Slit guidance ligand 3	Anterior midline diaphragmatic hernia	–
*Lox*	Lysyl oxidase	Central diaphragmatic hernia, thin diaphragm muscle	Lung hypoplasia, abnormal acini, abnormal elastic fibers
*Pdgfra*	Platelet-derived growth factor receptor, α-polypeptide	Posterolateral diaphragmatic hernia	Lung hypoplasia, abnormal alveoli, increased cell proliferation
*Robo1/2*	Roundabout guidance receptor 1/2	Posterior diaphragmatic hernia	Abnormal alveoli, thick septa
*Ndst1*	*N*-deacetylase-*N*-sulfotransferase-1	Diaphragmatic hernia in the anterior midline of the septum transversum	–

### Retinoid signaling pathway

Several knockout models have originated from gene pathways found to be associated with CDH such as the retinoid signaling pathway [13]. Mice deficient in both subtypes of *retinoic acid receptors α* and *β* (*Rarα* and *Rarβ*) have been shown to produce offspring with CDH [16–21], consistent with the vitamin A-deficient mouse models observed by Anderson [22, 23]. Single *Rar* null mutation mice did not exhibit the expected anomalies, which were reported in vitamin A-deficient rats [9]. However, when the function of these receptors was suppressed, multiple congenital anomalies were observed, including right-sided CDH in *Rarαβ2* mutant mice and left-sided CDH in *Rarαβ2*^+*/*−^ animals. In addition, these mice suffer from severe pulmonary hypertension at birth [9]. Unfortunately, these animals demonstrate a relative low rate of diaphragmatic defects and a high incidence of comorbidities including cranial, vertebral, limb, cardiac, foregut and pulmonary malformations that do not accurately reflect human CDH [20, 21]. Nevertheless, mutations in the *stimulated by retinoic acid gene 6* (*STRA6*) and *cellular retinoic acid binding protein 1* (*CRABP1*) on chromosome 15 have been identified in CDH patients [9].

#### *Nr2f2* (*Couptf2*)

Another gene associated with the retinoid signaling pathway is *chickovalbuminupstreampromoter* *transcription factor* *II* (*COUP*-*TFII*), a transcription factor belonging to the steroid/thyroid hormone receptor superfamily, whose DNA-binding site has been found to downregulate hormonal induction of retinoic acid receptors [24–26]. Recently, *COUP*-*TFII* was renamed as *nuclear receptor subfamily 2, group F, member 2 (NR2F2)* [13]. Mapping to chromosome 15q26, the *NR2F2* gene is located on a well-known CDH hotspot region in humans, making it a strong candidate gene for CDH pathogenesis. Based on this knowledge, You et al. [27] have developed a tissue-specific *Nr2f2* null mutant mouse model that exhibits Bochdalek-type CDH. In ablating *Nr2f2* in the foregut mesenchyme, diaphragmatic defects occurred due to the failure of the posthepatic mesenchymal plate to attach to the lateral body wall [27].

#### *Wt1*

Developing transgenic animal models for various applications has also uncovered genes less likely to be associated with CDH. Originally created as a model to study early urogenital development [28], *Wilm’s tumor 1 (Wt1)* null mutant mice displayed diaphragmatic defects in addition to urogenital malformations. Mutations of the *WT1* gene, which encodes a DNA-binding protein with four zinc fingers, have been identified in two CDH-related cases of Denys-Drash syndrome [29] and in one case of Meacham syndrome [30]. A decade ago, vitamin A-deficient, nitrofen and *Wt1* null mutant mouse models of CDH all suggested a common pathogenic mechanism for CDH development with similarities to the human condition [31]. Moreover, Carmona et al. [32] have shown that conditional deletion of *Wt1* in the septum transversum mesenchyme causes CDH in mice. It is now established that *Wt1* and *Couptf2* both interact with retinoid signaling during embryogenesis [33]. Interestingly, *Wt1* and *Couptf2* are not expressed in the muscle precursors but in the non-muscle mesenchymal component of the pleuroperitoneal folds (PPFs) [33].

#### *Msc* (*MyoR*) and *Tcf21* (*capsulin*)

The double mutant *musculin* (*Msc*)^−*/*−^ and *transcription factor 21* (*Tcf21*)^−*/*−^ mouse model, initially generated to characterize facial muscle development, features posterior CDH along with facial muscle defects. Although these double mutant mice died shortly after birth, the model’s diaphragmatic defect suggests that both *Msc* and *Tcf21* are required to maintain the integrity of the forming diaphragm. Today, their synonyms are known as *myogenic bHLH transcription factor R (MyoR)* and *capsulin*, respectively [34, 35].

#### *Gli2/3, Shh* and *Kif7*

*GLI*-*Kruppel* *family member 2 (Gli2)*, *Gli3* and *sonic hedgehog* (*Shh*) are members of a highly conserved morphogenetic family, which is known as the *Shh* signaling pathway [9, 36]. Murine models of VACTERL (vertebral, anorectal, cardiac, tracheoesophageal, renal and limb anomalies) syndrome involving *Gli2*^−*/*−^-, *Gli3*^−*/*−^- and *Gli2*^−*/*−^/*Gli3*^+*/*−^-mice developed left-sided CDH in addition to the reported VACTERL anomalies [36]. This is the first model that imitates the human VACTERL association, suggesting that aberations in *Shh* signaling might be involved in the VACTERL syndrome [37]. Furthermore, as *Gli2*, *Gli3* and *Wt1* all encode zinc finger proteins, other zinc finger transcription factors have also been associated with the development of transgenic animal models of CDH. *Kinesin family member 7 (Kif7)* has recently been identified as an essential component of the *Shh* signaling pathway as a negative regulator in early embryonic development [38]. *Kif7* encodes a motor protein that functions downstream of the G protein-coupled transmembrane receptor smoothened, and interacts with *Gli2* and *Gli3* [38]. Additionally, *Kif7* is required for the patterning and differentiation of the diaphragm in a model of syndromic CDH [38].

#### *Zfpm2*, *Gata4*, *Sox7* and *Frem1*

*Zinc finger protein, multitype 2 (ZFPM2)*, previously known as *friend of GATA*-*binding protein 2 (FOG2)*, similarly encodes a zinc finger protein that primarily interacts with another zinc finger protein *GATA4* to regulate various developmental processes in the diaphragm, lung and heart [39–41]. Moreover, *ZFPM2* is located on chromosome 8p23 and interacts physically with *COUP*-*TFII* [42, 43]. Despite sufficient evidence that certain genes are involved in the pathogenesis of different types of CDH, only one mutation in *ZFPM2* has been demonstrated in a single patient with non-syndromic CDH until now [9]. In a cohort of 275 CDH patient exomes, Longoni et al. [44] have estimated the prevalence of damaging *ZFPM2* mutations to be almost 5%. Furthermore, genetic analysis of a multi-generational family identified a heritable intragenic *ZFPM2* deletion with an estimated penetrance of 37.5% [44]. Mice treated with the chemical mutagen *N*-ethyl-*N*-nitrosourea in turn produced *Fog2*^−/−^ offspring with pulmonary hypoplasia and abnormal diaphragmatic development characteristic of CDH [39], while a double knockout strain of *Gata4* mice predisposed inbred mice to comparable physical defects [45]. Using mouse genetics, Merrell et al. [46] have confirmed that *Gata4* mosaic mutations in PPF-derived muscle connective tissue fibroblasts result in the formation of localized amuscular regions that are biomechanically weaker and lead to CDH. Both *ZFPM2* and *GATA4* genes have been reportedly deleted in human CDH patients [40], strengthening their claims as candidate genes for CDH. Recurrent microdeletions of 8p23.1 that include *GATA4* and *sex determining region Y*-*box 7 (SOX7)* are associated with a high risk of both CDH and cardiac defects [47]. Although *Gata4*-deficient mice showed both CDH and cardiac defects, humans with cardiac defects attributed to *GATA4* mutations have not been reported to have CDH [47]. On the other hand, Wat et al. [47] revealed that haploinsufficiency of *Sox7* or *Gata4* is sufficient to produce anterior CDH in mice and that haploinsufficiency of *SOX7* and *GATA4* may each contribute to the development of CDH in patients with 8p23.1 deletions. After identification of a *FRAS1*-*related extracellular matrix 1 (FREM1)* deletion in a child with isolated left-sided posterolateral CDH that was covered by a membranous sac, Beck et al. [48] created a *Frem1*-deficient mouse model that exhibits a similar phenotype including a retrosternal diaphragmatic defect and decreased levels of cell proliferation in the anterior portion of the developing diaphragm, demonstrating that *FREM1* deficiency can cause CDH in both humans and mice. Due to phenotypic overlaps between *Frem1*-deficient mice and mice that are deficient for the retinoic acid-responsive transcription factor *Gata4*, the authors performed further experiments, proving that *Frem1* interacts genetically with *Gata4* in the development of lung lobulation defects in this model [49].

#### *c-Met* and *Fgf10*

In addition to *ZFPM2* and *GATA4*, several other candidate genes have been implicated in CDH pathogenesis, yielding more transgenic animal models and thus greater insight into CDH etiology. For instance, the *mesenchymal*-*epithelial transition factor (c-Met)* proto-oncogene codes for a receptor tyrosine kinase responsible for migration of muscle precursor cells into the diaphragm [50], while *fibroblast growth factor 10 (Fgf10)* is critical for early lung development [51]. Oral administration of nitrofen in *Met*^−*/*−^ mice with amuscular diaphragms and *Fgf10*^−*/*−^ mice with hypoplastic lungs induced CDH in both murine models, suggesting that diaphragmatic defects occur independent of myogenesis and lung formation [52].

#### *Slit3*, *Lox*, *Pdgfra*, *Robo1/2* and *Ndst1*

As part of the *Slit guidance ligand (SLIT)* family of proteins responsible for axon crossing at the midline, homozygous *Slit3*-deficiency in mice causes failure of the central tendon region of the diaphragm to separate from the liver tissue, producing central (i.e. septum transversum) CDH [53, 54]. As a result, this specific model suffers from having the diaphragm defect in the ventral midline portion of the central tendon rather than in the posterolateral diaphragm. Other anomalies include renal and ureteric agenesis along with constant herniation of the liver and the gallbladder [54], which are uncommon in human CDH. Similarly, lysyl oxidase (Lox)^−*/*−^-mice were shown to have ruptured diaphragms due to fragmentation in the central tendon [55, 56]. On the other hand, while mice homozygous for null mutations in *platelet-derived growth factor receptor α* (*Pdgfra*) develop posterolateral diaphragmatic defects, they also present with a wide range of additional comorbidities including cardiac defects, renal and urogenital anomalies, facial clefts as well as pulmonary hypertension [57]. *Roudabout* (*Robo*) genes encode cell-surface receptors that have responsibility of their secreted ligands, Slit proteins, in various cellular processes [58–60]. The *Slit*-*Robo* signaling pathway has been demonstrated to play several key roles including neural crest cell migration and sensory ganglia morphogenesis, leukocyte chemotaxis, epithelial adhesion, embryonic and heart development as well as diaphragm and kidney formation [53, 54, 58, 61–65]. In fact, inactivation of *Robo1* and *Robo2* in mice leads to diaphragmatic malformation and mispositioning of the stomach in the thoracic instead of the abdominal cavity, which likely contributes to poor lung inflation and lethality at birth, reminiscent of CDH cases in humans [58]. Zhang et al. [66] have reported that ablation of the heparan sulfate biosynthetic enzyme *N*-deacetylase-*N*-sulfotransferase-1 (Ndst1) in mouse endothelium disrupts vascular development in the diaphragm, leading to hypoxia as well as subsequent diaphragmatic hypoplasia and central-type CDH. Interestingly, the phenotypes displayed in these mice resembled the developmental defects observed in *Slit3* knockout mice. Furthermore, introduction of a heterozygous mutation in *Robo4*, the gene encoding the cognate receptor of Slit3, aggravated the defects in vascular development in the diaphragm and CDH [66]. Consequently, these results suggest that loss of *Ndst1* causes defective diaphragm vascular development and CDH and that heparan sulfate facilitates the angiogenic *Slit3*-*Robo4* signaling cascade during vascular development.

## Conclusion and future directions

Experimental animal models of CDH have not only allowed us to study the pathophysiology and etiology of this relatively complex birth defect, but have also provided new insights into the molecular and biochemical basis, thus contributing to advances in the medical and surgical management. Hence, CDH animals in which this malformation occurs naturally are ideal models to investigate disease pathogenesis and associated pulmonary hypoplasia, as there is little or no interference to the animal prior to the study. Additionally, transgenic animal models of CDH not only mimic the natural occurrence of this condition, but also give a better understanding into the genes involved and how their modification might alter the course of the disease. Teratogen-induced CDH models although useful, have in turn the drawback of exposing the animals to a generalized noxious stimulus, which can result in widespread detrimental effects rather than simply targeting a specific organ system. The combination of transgenic animal models with regenerative tissue engineering and stem cell-based therapy may play a role in future CDH research by developing a myogenic patch capable of restoring muscle fraction in fetal diaphragmatic defects and promoting regeneration of hypoplastic lungs [67–70].
